# Shaped by leaky ER: Homeostatic Ca^2+^ fluxes

**DOI:** 10.3389/fphys.2022.972104

**Published:** 2022-09-07

**Authors:** Annemarie Schulte, Robert Blum

**Affiliations:** ^1^ Department of Neurology, University Hospital of Würzburg, Würzburg, Germany; ^2^ Department of Anesthesiology, Intensive Care, Emergency Medicine and Pain Therapy, University Hospital of Würzburg, Würzburg, Germany

**Keywords:** Ca^2+^ homeostasis, Ca^2+^ ion analysis, ER Ca^2+^ store, ER Ca^2+^ imaging, store-operated Ca^2+^ entry, Ca^2+^ leak, SERCA, Ca^2+^ oscillation

## Abstract

At any moment in time, cells coordinate and balance their calcium ion (Ca^2+^) fluxes. The term ‘Ca^2+^ homeostasis’ suggests that balancing resting Ca^2+^ levels is a rather static process. However, direct ER Ca^2+^ imaging shows that resting Ca^2+^ levels are maintained by surprisingly dynamic Ca^2+^ fluxes between the ER Ca^2+^ store, the cytosol, and the extracellular space. The data show that the ER Ca^2+^ leak, continuously fed by the high-energy consuming SERCA, is a fundamental driver of resting Ca^2+^ dynamics. Based on simplistic Ca^2+^ toolkit models, we discuss how the ER Ca^2+^ leak could contribute to evolutionarily conserved Ca^2+^ phenomena such as Ca^2+^ entry, ER Ca^2+^ release, and Ca^2+^ oscillations.

## Introduction

The tight control of coordinated homeostatic calcium ion (Ca^2+^) fluxes is of fundamental importance for cellular signaling and health (Berridge et al., 2003). Evolutionary conserved mechanisms maintain Ca^2+^ levels in every cell type, while different adaptions modulate cell type-specific functions. The major intracellular Ca^2+^ store is the endoplasmic reticulum (ER) (Verkhratsky, 2005). Despite its central role in the pathophysiology of many severe diseases (Mekahli et al., 2011), our understanding of how ER Ca^2+^ fluxes shape cellular Ca^2+^ signaling is still poor. One reason is that Ca^2+^ signals are typically monitored in the cytosol. With the improvement of ER Ca^2+^ imaging techniques, it became technically possible to directly monitor Ca^2+^ dynamics in the ER, with reasonably good spatiotemporal resolution (Rehberg et al., 2008; Samtleben et al., 2013; Rodriguez-Garcia et al., 2014; de Juan-Sanz et al., 2017; Schulte et al., 2022). These experiments unraveled a surprisingly pronounced physiological role of the ER Ca^2+^ leak in shaping Ca^2+^ signals (Thastrup et al., 1989; Camello et al., 2002; Flourakis et al., 2006; Samtleben et al., 2015; Lemos et al., 2021).

## Principles and limitations of ER Ca^2+^ imaging

In the cytosol, the resting Ca^2+^-concentration is about 100 nM. Upon stimulation, cytosolic Ca^2+^ concentrations rise to 0.5–1 µM and can reach tens of micromolar close to active Ca^2+^-channels (Bootman and Bultynck, 2020). In the ER lumen, Ca^2+^ concentrations are in the range of ∼50 µM up to 1 mM. For this reason, ER Ca^2+^ indicators need a low affinity for Ca^2+^ [dissociation constant (K_d_) ∼100–200 µM] while maintaining high responsiveness to Ca^2+^.

For ER Ca^2+^ imaging, three fundamentally different principles were developed. One is based on the direct loading of cells with synthetic acetoxymethyl (AM)-estered Ca^2+^ indicators, such as Mag-Fura2-AM [Kd ∼ 25–50 µM (Hofer and Machen, 1993; Solovyova and Verkhratsky, 2002)], Mag-Fluo4 [K_d_ ∼ 22 μM (Laude et al., 2005)] or Fluo5N [∼90 μM (Chen et al., 2015)]. The technique is well-suited for some cell types; however, a certain amount of the indicator becomes reactive in the cytosol, thereby causing ‘mixed’ ER-cytosol signals. To remove undesirable indicator from the cytosol, cells can be permeabilized (e.g., with digitonin or streptolysin) or dialyzed with the help of a patch-clamp pipette (Solovyova and Verkhratsky, 2002). In permeabilized cells, Mag-Fluo4 leaking out of the ER can also be quenched with an antibody (Rossi and Taylor, 2020). Surprisingly, after quenching, Mag-Fluo4-AM showed a much higher K_d_^Ca2+^-value in the ER (∼1 mM instead of 22 µM), most likely due to incomplete de-esterification in the ER lumen (Rossi and Taylor, 2020).

A strategy to accumulate synthetic Ca^2+^ indicators in the ER lumen of non-disrupted cells is targeted esterase-induced dye loading (TED) (Rehberg et al., 2008; Samtleben et al., 2013). For TED, a genetically overexpressed carboxylesterase hydrolyses a synthetic low-affinity acetoxymethyl (AM) ester in the ER lumen, thereby forming a hydrophilic dye/Ca^2+^ complex. The fluorescent Ca^2+^-dye complex is trapped and enriched in the ER lumen and provides an excellent signal-to-noise ratio (Rehberg et al., 2008). The best available indicator for TED is still Fluo5N-AM (Samtleben et al., 2013). The de-estered, Ca^2+^-sensitive form of Fluo5N is detectable in the ER for hours. In the cytosol, Fluo5N is reactive but barely visible. Unfortunately, Fluo5N is extremely light sensitive (bleaching and random flashing), making it difficult to image Fluo5N/Ca^2+^ complexes (Samtleben et al., 2013; Schulte et al., 2022).

The third strategy is based on ER-targeted low-affinity GECIs (genetically encoded Ca^2+^ indicator) such as the D1ER-derivate D4ER (Kipanyula et al., 2012), CEPIAer (Suzuki et al., 2014), ER-GCaMP6-150/210 (de Juan-Sanz et al., 2017) or ER-GAP-derivates (Rodriguez-Garcia et al., 2014; Alonso et al., 2017). For GECIs, high expression levels using a strong vector promoter are required to achieve an appropriate GECI signal. This increases the risk of protein misfolding or mistargeting by saturating the ER translocation and ER retention and retrieval processes.

We recently compared TED using Fluo5N with the GECI ER-GCaMP6-150. The data showed that TED is well suited to visualize fast Ca^2+^ signal onsets (Schulte et al., 2022). ER-GCaMP6-150 showed excellent on-off rates, was quite bleach resistant and allowed imaging for up to 1 h on the same cells (Schulte et al., 2022). In all our direct ER imaging experiments, ‘typical’ excitation light conditions could stop ongoing ER Ca^2+^ oscillations within ∼2 min (Schulte et al., 2022), albeit the indicators themselves were still reactive. We observed the phenomenon, loss of reactivity, in all types of cells we ever investigated (Hek293, HeLa, BHK21, astrocytes, neurons). We do not have an explanation for this observation. Hence, extreme low excitation light conditions might be needed for all ER Ca^2+^ imaging experiments as the light sensitivity of ER Ca^2+^ dynamics might be of biological and not methodological origin.

Nowadays, for dual-color Ca^2+^ imaging (ER/cytosol), a green-fluorescent ER Ca^2+^ indicator and a red fluorescent cytosolic dye are a good combination (Rodríguez-Prados et al., 2020; Schulte et al., 2022). We recommend using a GECI, such as ER-GCaMP6-150/210, with AM-ester based loading of the cytosolic dye Cal-590 (Birkner and Konnerth, 2019; Schulte et al., 2022). Fluorescence of both dyes can be well separated with standard fluorescence microscopy. The excellent signal-to-noise ratio of Cal-590 allows low-light illumination conditions and does not destroy ER Ca^2+^ dynamics (Schulte et al., 2022).

## Is there a defined ‘resting’ ER Ca^2+^ concentration?

In physiology, the extracellular and cytosolic ion concentrations are well defined. This is not true for the ER Ca^2+^ concentration. Resting ER Ca^2+^ levels were described to be in the range of 50 µM up to 1 mM; meaning a difference factor of ×20. Depending on cell type, indicator, or calibration approach, resting ER Ca^2+^ concentrations range between 60 and 270 µM in cultured sensory neurons (Solovyova et al., 2002), 700–800 µM in HeLa and Hek293 cells (Tang et al., 2011), ∼150 µM in primary hippocampal neurons (de Juan-Sanz et al., 2017), and ∼400 µM in cultured astrocytes (Rodríguez-Prados et al., 2020).

It is not easy to determine the exact ‘resting’ ER Ca^2+^ concentration in living cells. The ER Ca^2+^ range can be estimated in permeabilized [‘leaky cells’ (Streb et al., 1983)] or disrupted [‘whole-cell patch clamp (Solovyova et al., 2002)] conditions, after blockade of the SERCA. Standardized Ca^2+^ calibration solutions (zero Ca^2+^ to ∼2–5 mM free Ca^2+^) are applied extracellularly until an equilibrium state is formed between the extracellular space, the cytosol, and the ER lumen. However, permeabilized cells are no longer in a physiological state, and potential loss of small-molecule Ca^2+^ dyes may confound Ca^2+^ calibration. Also, it is difficult to provide a ‘resting’ ER Ca^2+^ level for living cells with unknown Ca^2+^ toolkit and physiological state.

We imaged an entire calibration process for ∼20 min with a temporal resolution of 5 Hz (Schulte et al., 2022). The data confirmed that Ca^2+^ concentration in the ER lumen is about thousand-fold higher than in the cytosol and about 10–20x times lower than in the extracellular space (Schulte et al., 2022). Notably, the ER Ca^2+^ store can be rapidly refilled, within seconds, when the SERCA is blocked (Schulte et al., 2022). It can well be that this fast passive ER refilling in permeabilized cells is a ‘calcium tunnelling’ phenomenon (Petersen et al., 2017) and occurs through the ER Ca^2+^ leak channels.

## The ER Ca^2+^ leak, a surprisingly strong intracellular Ca^2+^ flux

In a simplified view, a living cell generates the resting membrane potential by potassium ions that leak from inside the cell to the outside, via K^+^ ‘leak’ channels. The driving force of the potassium gradient is maintained by the high energy consuming Na^+^/K^+^ ATPase. The fundamental principle is similar for Ca^2+^ fluxes from the ER Ca^2+^ store to the cytosol. A very high force drives the Ca^2+^ from the ER lumen through the ER Ca^2+^ leak channels into the cytosol, an effect which might be electrogenic (Burdakov et al., 2005; Verkhratsky, 2005). The molecular identity of the ER Ca^2+^ leak is not entirely clear (Lemos et al., 2021), but there is strong experimental evidence that the Sec61 translocon complex is one of the main mediators of passive ER Ca^2+^ leak (Flourakis et al., 2006; Schäuble et al., 2012). Sec61 complexes are evolutionarily highly conserved, are ubiquitous, and transcriptome data revealed that they are expressed at very high levels. Sec61 complexes are non-redundant proteins involved in protein synthesis (Lang et al., 2017), meaning that a cell cannot fully avoid ER Ca^2+^ leak. To maintain ER Ca^2+^ levels, high activity of the SERCA (sarcoplasmic/endoplasmic reticulum Ca^2+^ ATPase) is needed.

An easy way to unmask the ER Ca^2+^ leak is by blocking the SERCA, irreversibly with the drug thapsigargin (Thastrup et al., 1989), or acutely with CPA (cyclopiazonic acid) (Samtleben et al., 2015). The rate at which SERCA blockade empties the ER can vary widely from cell to cell. Direct ER Ca^2+^ imaging, however, suggests that the ER Ca^2+^ leak is a strong, temperature-dependent, persistent intracellular Ca^2+^ flux. In neurons, for instance, SERCA blockade with CPA depletes the ER Ca^2+^ store within 1–2 min (Samtleben et al., 2015; de Juan-Sanz et al., 2017). In cultured astrocytes, acute application of SERCA blocking agents [thapsigargin (Schulte et al., 2022) or tBHQ (Rodriguez-Prados et al., 2020)] caused a drop in the fluorescence signal (F) from F_rest_ to F_min_ in about a minute.

Ca^2+^-imaging in the cytosol is not well-suited to investigate the spatio-temporal dynamics of the ER Ca^2+^ leak. In neurons, in presence of extracellular Ca^2+^, SERCA blockade induces Ca^2+^ entry over the plasma membrane, thus masking the contribution of the ER Ca^2+^ leak to the cytosolic Ca^2+^ transient. In Ca^2+^-free extracellular solution, the expected cytosolic Ca^2+^ signal is often barely detectable, because both the ER and cytosolic Ca^2+^ are rapidly lost to extracellular sites (Samtleben et al., 2015).

## The ER Ca^2+^ leak triggers homeostatic Ca^2+^ fluxes

**FIGURE 1:**
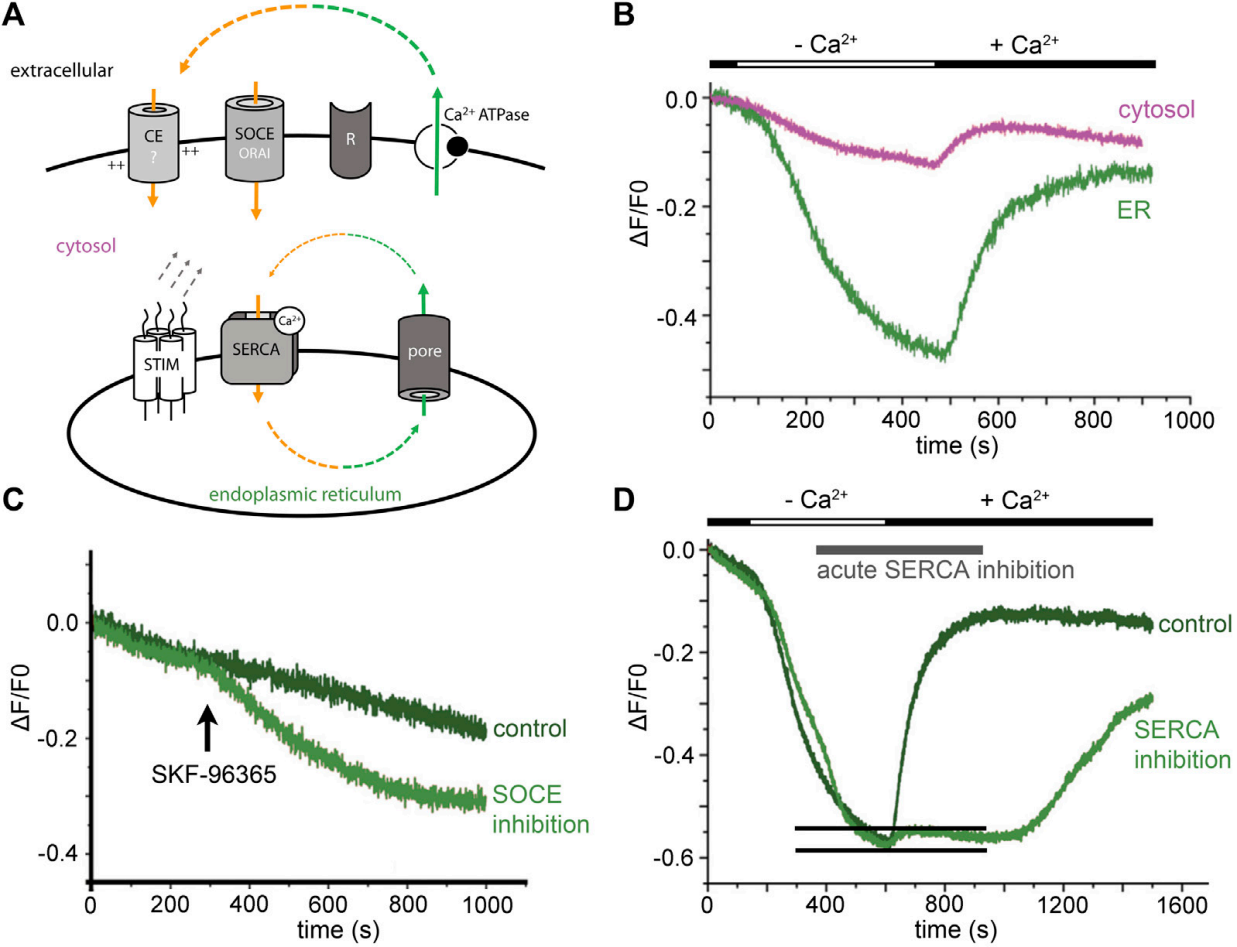
ER Ca^2+^ leak in neurons triggers resting Ca^2+^ entry. **(A)** Minimal model: ER Ca^2+^ leak and cellular Ca^2+^ loss (green) need to be counterbalanced from extracellular sides (orange). Activity of the SERCA closes the Ca^2+^ loop. Leaking Ca^2+^ is partly rescued from the cytosol by the SERCA, but this cannot prevent ER Ca^2+^ depletion in Ca^2+^-free solution. The best candidates for homeostatic Ca^2+^ influx are ORAI channels and yet unknown Ca^2+^ entry (CE?) mediators. **(B)** During extracellular Ca^2+^ withdrawal, ER Ca^2+^ levels start to drop within seconds. Extracellular Ca^2+^ is needed to counterbalance homeostatic ER Ca^2+^ loss. **(C)** Inhibition of Ca^2+^ entry by short application of the SOCE-inhibitor SKF-96365 (25 µM) reduces ER Ca^2+^ levels. **(D)** ER Ca^2+^ refilling with and without acute SERCA blockade. A short, transient ER Ca^2+^ signal is observed in presence of the SERCA inhibitor (indicated by two black lines). The original experiments were performed in primary hippocampal neurons, during neuronal activity blockade with 100 nM tetrodotoxin. Cells were not stimulated via any receptor (R). Cytosolic Ca^2+^ signals were measured with Oregon Green 488 BAPTA-1 and are shown in magenta. TED with Fluo5N-AM were used for direct ER Ca^2+^ imaging (green lines in **A**–**C**). [Modified according to (Samtleben et al., 2015)].

Existence of an evolutionarily conserved ER Ca^2+^ leak raises the question of its influence on homeostatic Ca^2+^ fluxes (minimal model in Figure 1A). When we blocked neuronal activity of hippocampal neurons and removed extracellular Ca^2+^ acutely, the ER Ca^2+^ signal dropped to a F_min_ plateau signal within a few minutes (Figure 1B) (Samtleben et al., 2015). Subsequent acute addition of CPA did not further reduce the ER Ca^2+^ signal. Similarly, metabotropic ER Ca^2+^ release was abolished in cerebellar Purkinje cells that were kept in Ca^2+^-free solution for some minutes, as shown with cytosolic Ca^2+^ imaging (Hartmann et al., 2014). Removal of extracellular Ca^2+^ appear to empty the ER Ca^2+^ store virtually completely in some minutes.

Evidently, passive ER Ca^2+^ loss cannot be restored from cytosolic calcium by SERCA activity alone. Hence, a constitutively active resting Ca^2+^ entry is needed to maintain ER Ca^2+^ levels. The phenomenon in which depletion of the intracellular Ca^2+^-store activates Ca^2+^ influx is called store-operated Ca^2+^ entry (capacitive Ca^2+^ entry) (Putney et al., 2017). We tested SOCE-blockers to find out whether a constitutively active calcium entry mechanism compensates passive ER Ca^2+^ loss. The data confirmed that acute application of SOCE-blockers (SKF-96365/BTP-2) induces an immediate drop in ER Ca^2+^ levels (Figure 1C) (Samtleben et al., 2015). Thus, resting Ca^2+^ influx over the plasma membrane exists and is, in the end, triggered by the ER Ca^2+^ leak and maintained by SERCA activity (summarized in Figure 1A).

The resting Ca^2+^ influx is functionally relevant as it is a distinct mechanism for regulating gene expression (Lalonde et al., 2014) and seem to also trigger local Ca^2+^ influx events, so-called ‘signal-close-to-noise Ca^2+^ activity’ (Prada et al., 2018). The Ca^2+^ toolkit underlying homeostatic Ca^2+^ influx mechanisms is not well known (Figure 1A) but in hippocampal neurons, it is resistant to an inhibitor cocktail containing TTX (for voltage-gated sodium channels), APV and CNQX (to block ionotropic glutamate receptors), and Ni^2+^-ions (to reduce activity of low-threshold activated VGCCs) (Prada et al., 2018). We think that constitutive active ORAI channels contribute to ER-leak-triggered, homeostatic Ca^2+^ influx (Figure 1A).

Why are neurons or astrocytes investing so much energy in maintaining homeostatic Ca^2+^ fluxes via the extracellular space? Perhaps, resting Ca^2+^ fluxes are needed to signal neuronal health. More SOCE-like Ca^2+^ entry or less active removal of cytosolic Ca^2+^ would lead to cellular Ca^2+^ overload. This has clinical implications. For instance, when SOCE blockers are used to prevent acute or neurodegenerative Ca^2+^ overload, resting homeostatic Ca^2+^ influx would be reduced. This would also reduce ER Ca^2+^ levels and thereby induce ER stress signaling (Mekahli et al., 2011) and mitochondrial dysfunction (Garbincius and Elrod, 2022).

## SERCA-independent ER refilling

Theoretically, fast passive Ca^2+^ influx from the extracellular side might be enough to locally refill the ER lumen. In a direct ER Ca^2+^ imaging experiment, we emptied the ER Ca^2+^ store of neurons in Ca^2+^-free solution (Figure 1D). When we re-added extracellular Ca^2+^ and blocked the SERCA acutely, a short, transient increase in ER Ca^2+^ levels was observed (Samtleben et al., 2015). We cannot exclude incomplete block of the SERCA in this experiment. However, the transient-like character of the signal suggests that Ca^2+^ enters the ER passively and is then lost through the ER Ca^2+^ leak. Future developments in life-cell imaging combined with super-resolution techniques might solve the question whether there are regulated ‘tunnel-like’ microdomains between the ER lumen and the extracellular space. General models for Ca^2+^ tunnelling mechanisms are discussed since many years (Petersen et al., 2017). It can well be that ER Ca^2+^ sparks (Cheng and Lederer, 2008) depend on such a ‘tunnel-like’ microdomain. Proximity of ‘leaky’ ER microdomains, ORAI, and Stim complexes might be a minimal requirement for electrogenic, local Ca^2+^ signals. The fluxes should be sufficient to trigger voltage-dependent Ca^2+^ influx as well as local induction of Ca^2+^-induced Ca^2+^ release (Ca^2+^-iCR) (see later).

## Shaping of Ca^2+^ fluxes by the ER Ca^2+^ leak: Clues from cultured astrocytes

In our recent work, we used cultured cortical astrocytes (mouse) and dual color Ca^2+^ imaging (ER/cytosol) to find out how ER Ca^2+^ dynamics shape homeostatic Ca^2+^ fluxes. Astrocytes are well suited as a prototypical Ca^2+^ model (Verkhratsky and Nedergaard, 2018; Lim et al., 2021). The cells are very responsive and can be cultured with high purity, which makes it easy to determine their Ca^2+^ toolkit, e.g., with RNA-seq (Hasel et al., 2017; Schulte et al., 2022). The cell model expresses two IP_3_ receptors (*Itpr1* and *Itpr2*), but no ryanodine receptors. Notably, there is just one SERCA (SERCA2, *Atp2a2*) and a high amount of plasma membrane Ca^2+^-ATPases (*Atp2b1, Atp2b4*). Sec61a and other ER Ca^2+^ leak channel candidates (e.g., Tmco1, presenilin) are highly expressed (Schulte et al., 2022). Based on transcriptome data and analysis of calcium signal profiles observed in individual cells, minimal models can be designed to discuss how the ER Ca^2+^ leak could shape Ca^2+^ signals.

### The ER Ca^2+^ leak is interlinked with Ca^2+^-induced Ca^2+^ release

**FIGURE 2:**
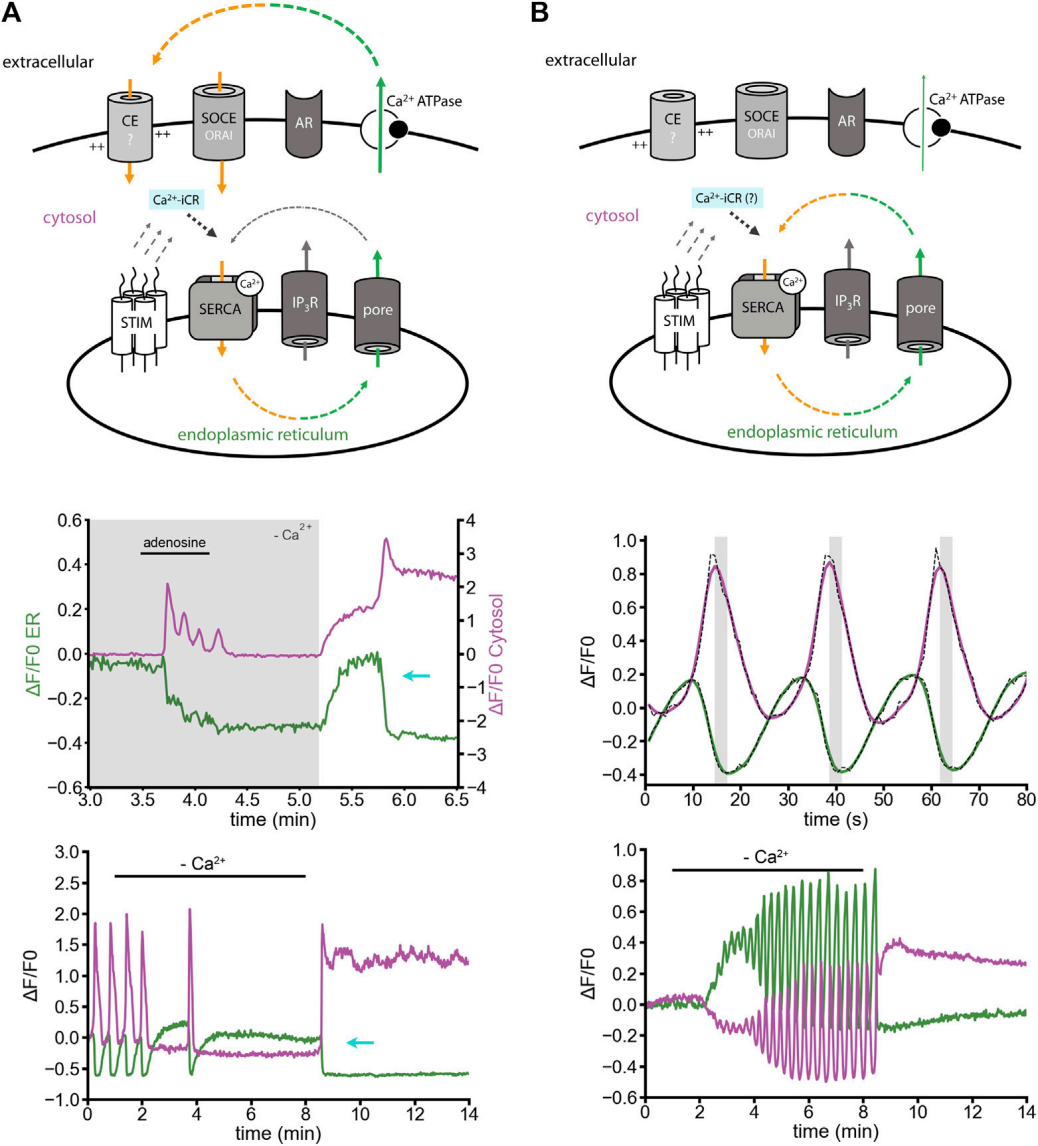
Induced ER Ca^2+^ release versus ER Ca^2+^ oscillation in cultured astrocytes. Simultaneous imaging of ER and cytosolic Ca^2+^ with the indicators: ER-GCaMP6-150 (green) and Cal-590-AM (magenta) in non-disrupted cells. **(A)** Minimal model: Ca^2+^-induced Ca^2+^ release (Ca^2+^-iCR, cyan) is induced by SOCE-like Ca^2+^ entry after ER replenishment. Middle panel: In Ca^2+^-free solution, adenosine-induced Ca^2+^ oscillations persist for about half a minute. Delayed Ca^2+^ replenishment induces a Ca^2+^-iCR phenomenon, in absence of ryanodine receptors (cyan arrow). Lower panel: Removal of extracellular Ca^2+^ and subsequent re-adding of extracellular Ca^2+^ can be sufficient to stimulate Ca^2+^-iCR. **(B)** (Minimal model) Spontaneous Ca^2+^ oscillation in Ca^2+^-free solution. (Middle panel) Spontaneous Ca^2+^ oscillations are shaped by a circular relationship between ER and cytosolic signals. Simultaneous imaging revealed a time lag of some seconds between peak signals in the cytosol and the ER Ca^2+^ release signal (grey). Raw traces (black dashed line) and the low-pass filtered traces (in color) are plotted. Lower panel: Spontaneous Ca^2+^ oscillation in Ca^2+^-free solution. The sarco-/endoplasmic reticulum Ca^2+^ ATPase (SERCA) recycles intracellular Ca^2+^. Candidates for ER Ca^2+^ leak are IP_3_ receptors and ER Ca^2+^ leak channels. Ca^2+^ released from the ER stays in the cell, is not exported to extracellular sides (compare with Figure 3), and the SERCA adapts its activity to the cytosolic Ca^2+^ concentration. [Modified according to (Schulte et al., 2022)].

Cultured cortical astrocytes show depolarization-dependent ER Ca^2+^ release (Rodríguez-Prados et al., 2020), but do not express ryanodine receptors (Hasel et al., 2017; Schulte et al., 2022). Surprisingly, ER Ca^2+^ refilling by Ca^2+^ entry can induce Ca^2+^-iCR, which empties the ER Ca^2+^ store again, and keeps cytosolic Ca^2+^ levels high (Figure 2A) (Schulte et al., 2022). In astrocytes, the effect was observed: 1) after adenosine-induced Ca^2+^ release in Ca^2+^-free solution and delayed re-adding of extracellular Ca^2+^ (Figure 2A, middle panel); 2) during ER replenishment by homeostatic Ca^2+^ entry after depleting the ER Ca^2+^ store in Ca^2+^-free solution (Figure 2A, lower panel; for neurons see Figure 1B). The effect might be mediated by IP_3_-receptors, or ER Ca^2+^ leak channels by a yet unknown mechanism. Returning to resting ER Ca^2+^ levels would then require cytosolic Ca^2+^ export to extracellular sides, e.g. by active transport via Ca^2+^-ATPases or secondary active transport mechanisms (Na^+^/Ca^2+^ exchanger).

### Is the ER Ca^2+^ leak the basic trigger for spontaneous Ca^2+^ oscillations?

Ca^2+^ oscillations in astrocytes can also appear spontaneously, without an obvious external stimulator. Ca^2+^ oscillations are often linked to changing speed of ER Ca^2+^ efflux, depending on IP_3_ receptor activity or IP_3_ metabolism (Dupont et al., 2011). In our view, increased IP_3_ levels are certainly a trigger of Ca^2+^ oscillations, though it is likely not maintaining the Ca^2+^ oscillation (Figure 2B). ER/cytosol Ca^2+^ signals during spontaneous Ca^2+^ oscillations are in a non-linear (circular) slope relationship with a spatiotemporal time-lag in the range of seconds (Schulte et al., 2022). Furthermore, oscillatory ER Ca^2+^ fluxes can go on for minutes, in presence and absence of extracellular Ca^2+^ (Figure 2B, lower panel) (Schulte et al., 2022).

We would like to suggest the following model: a passive ER Ca^2+^ leak pore, like Sec61a, maybe in concert with other passive ER Ca^2+^ leak mediators (Lemos et al., 2021), mediates a constant ER Ca^2+^ flux to the cytosol. The ER Ca^2+^ leak is powerful and fast enough to shape the spatiotemporal profile of the ER Ca^2+^ oscillations. The Ca^2+^ stays in the cell and the SERCA increases its activity depending on the cytosolic Ca^2+^ concentration. Thus, Ca^2+^-dependency of the SERCA2 (Satoh et al., 2011) might be sufficient to explain the oscillatory behavior of ER-Ca^2+^ influx and efflux (Figure 2B). The weakness of this model is that Sec61a, the Ca^2+^ leak channel that would best fit to the concept, is a highly regulated protein (Daverkausen-Fischer and Pröls, 2022). Still, the sum of all ER leak mechanisms could fulfill the fundamental property of a passive ER Ca^2+^ leak pore through which Ca^2+^ flow is rather fast, for instance as seen after SERCA inhibition. How the ER Ca^2+^ leak mechanisms are regulated, how it’s activity is reduced, enhanced, activated or blocked, remains to be understood.

Mitochondria are also handling Ca^2+^, show mitochondrial Ca^2+^ (_m_Ca^2+^) oscillations and are involved in the oscillation cycle, and might account for the temporal shift between ER and cytosolic calcium signals (Ishii et al., 2006; Lim et al., 2021). Much focus was put on the function of the mitochondrial Ca^2+^ uniporter complex (MCU complex) (Stefani et al., 2011). However, there is also MCU-independent Ca^2+^ uptake to mitochondria (Garbincius and Elrod, 2022). For instance, in MCU knockout cells, agonist-induced increase in _m_Ca^2+^ is strongly reduced, or even abolished (Young et al., 2022; Álvarez-Illera et al., 2020). However, _m_Ca^2+^ oscillations can be MCU-independent, at least in *C. elegans* (Álvarez-Illera et al., 2020). How mitochondria handle Ca^2+^ during Ca^2+^ oscillations, in response to stimuli or in cases of _m_Ca^2+^-overload or underload, might be analyzed with triple color imaging experiments. For such experiments, simultaneous Ca^2+^ imaging, e.g. ER Ca^2+^ in green, cytosolic Ca^2+^ in red and mitochondrial Ca^2+^ in far-red, need to be validated.

### The ER Ca^2+^-leak shapes agonist-induced Ca^2+^ fluxes

**FIGURE 3:**
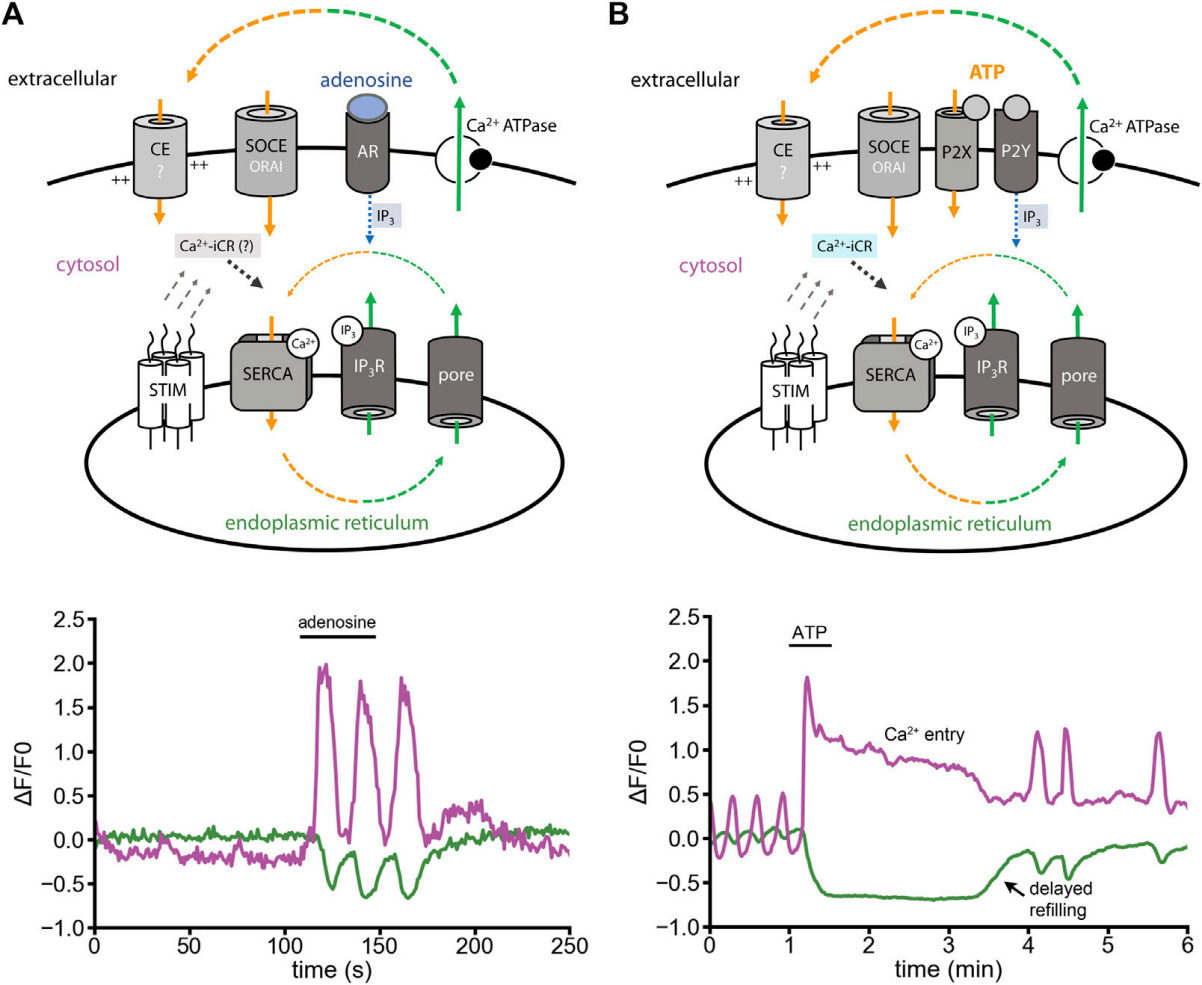
Adenosine- versus ATP-induced Ca^2+^ release in cultured astrocytes. Experiments were performed in presence of extracellular Ca^2+^. **(A)** Minimal model: Adenosine evokes IP_3_-iCR (IP_3_, blue). Ca^2+^ oscillations are induced by IP_3_-iCR. Ca^2+^ release and Ca^2+^ entry are in balance. Theoretically, Ca^2+^-iCR should contribute to the Ca^2+^ fluxes (indicated in grey). Lower panel: Oscillatory Ca^2+^ cycle induced by adenosine (10 µM) with cytosolic Ca^2+^ signals (magenta) and ER Ca^2+^ signals (green). **(B)** Minimal model: ATP binds to P2X and P2Y receptors. Metabotropic P2Y receptors evoke IP_3_-iCR (blue). P2X receptors might promote depolarizing Ca^2+^ influx, activation of voltage-gated Ca^2+^ channels and ER Ca^2+^ release through Ca^2+^-iCR (cyan). Lower panel: In response to ATP, the cytosolic Ca^2+^ increases while the ER Ca^2+^ decreases. Ca^2+^ entry is induced and creates a signal shoulder in the cytosol. The ER is not refilled during the Ca^2+^ entry shoulder. [Modified according to (Schulte et al., 2022)].

One widely studied Ca^2+^ signal is IP_3_-induced Ca^2+^ release (IP_3_-iCR). In astrocytes, IP_3_-iCR can be activated by adenosine via the highly expressed metabotropic receptor Adora1a (Figure 3A, minimal model). Fast adenosine stimuli activate fast ER Ca^2+^ release and subsequent Ca^2+^ oscillations (Figure 3A, lower panel) (Schulte et al., 2022). ATP, in contrast to adenosine, does not only evoke IP_3_-iCR through metabotropic P2Y receptors, but also opens ionotropic P2X receptors (Figure 3B, minimal model). When we stimulated astrocytes with ATP, a long-lasting cytosolic Ca^2+^ signal was induced (Figure 3B, lower panel). This Ca^2+^ entry shoulder did not contribute to ER refilling, but was likely preventing it (Schulte et al., 2022).

Why is the ATP-induced signal so different from exclusively metabotropic signals? The best explanation is that ATP activates a mixture of IP_3_-iCR and Ca^2+^-iCR (Figure 3B). Here, Ca^2+^-iCR would delay the refilling of the ER even though Ca^2+^ entry is ongoing (Figure 3B). In context of above-mentioned data (Figure 2), we think that a pronounced ER Ca^2+^ leak, and not only IP_3_-receptors, counteract ER refilling.

## The ER Ca^2+^ leak, a fundamental driver of homeostatic Ca^2+^ fluxes

Over the last years, our view on the ER Ca^2+^ leak has drastically changed. The process can no longer be seen as an ‘unavoidable’ side effect of protein translation or as a slow, passive intracellular Ca^2+^ flux. Data from neurons and astrocytes clearly show that resting ER Ca^2+^ leak is upstream of resting Ca^2+^ entry, and thereby indirectly responsible for resting Ca^2+^ levels in the cytosol and ER. The ER Ca^2+^ leak fundamentally shapes cellular Ca^2+^ signals and ER Ca^2+^ oscillations.

One of the most exciting questions for future research is how ER leak channels contribute to microdomain signaling [Ca^2+^-tunnelling (Petersen et al., 2017)], local Ca^2+^ sparks (Cheng and Lederer, 2008), and electrogenic effects for local cellular excitability (Burdakov et al., 2005). Genetically engineered voltage dyes, targeted to the inner-side of the ER-membrane, might help to address electrogenic effects of ER leak channels.

For clinical research, it will be important to know how the ‘Ca^2+^ overload’ phenomenon arises and contributes to mitochondrial dysfunction and cell damage (Mekahli et al., 2011; Garbincius and Elrod, 2022). It can well be that Ca^2+^ overload in the cytosol and in mitochondria is triggered by an increased ER Ca^2+^ leak (ER Ca^2+^ ‘underload’) that continuously promotes resting homeostatic Ca^2+^ influx.

## References

[B1] Alonso M. T. Rojo-Ruiz J. Navas-Navarro P. Rodriguez-Prados M. Garcia-Sancho J. (2017). Measuring Ca(2+) inside intracellular organelles with luminescent and fluorescent aequorin-based sensors. Biochim. Biophys. Acta. Mol. Cell Res. 1864 (6), 894–899. 10.1016/j.bbamcr.2016.12.003 27939433

[B28] Álvarez-Illera P. García-Casas P. Fonteriz R. I. Montero M. Alvarez J. (2020). Mitochondrial Ca(2+) dynamics in MCU knockout *C. elegans* worms. Int. J. Mol. Sci. 21 (22), E8622. 10.3390/ijms21228622 33207633 PMC7696937

[B2] Berridge M. J. Bootman M. D. Roderick H. L. (2003). Calcium signalling: Dynamics, homeostasis and remodelling. Nat. Rev. Mol. Cell Biol. 4 (7), 517–529. 10.1038/nrm1155 12838335

[B3] Birkner A. Konnerth A. (2019). Deep two-photon imaging *in vivo* with a red-shifted calcium indicator. Methods Mol. Biol. 1929, 15–26. 10.1007/978-1-4939-9030-6_2 30710264

[B4] Bootman M. D. Bultynck G. (2020). Fundamentals of cellular calcium signaling: A primer. Cold Spring Harb. Perspect. Biol. 12 (1), a038802. 10.1101/cshperspect.a038802 31427372 PMC6942118

[B5] Burdakov D. Petersen O. H. Verkhratsky A. (2005). Intraluminal calcium as a primary regulator of endoplasmic reticulum function. Cell calcium 38 (3-4), 303–310. 10.1016/j.ceca.2005.06.010 16076486

[B6] Camello C. Lomax R. Petersen O. H. Tepikin A. V. (2002). Calcium leak from intracellular stores--the enigma of calcium signalling. Cell calcium 32 (5-6), 355–361. 10.1016/s0143416002001926 12543095

[B7] Chen M. Van Hook M. J. Thoreson W. B. (2015). Ca^2+^ diffusion through endoplasmic reticulum supports elevated intraterminal Ca^2+^ levels needed to sustain synaptic release from rods in darkness. J. Neurosci. 35 (32), 11364–11373. 10.1523/JNEUROSCI.0754-15.2015 26269643 PMC4532764

[B8] Cheng H. Lederer W. J. (2008). Calcium sparks. Physiol. Rev. 88 (4), 1491–1545. 10.1152/physrev.00030.2007 18923188

[B22] Daverkausen-Fischer L. Pröls F. (2022). Regulation of calcium homeostasis and flux between the endoplasmic reticulum and the cytosol. J. Biol. Chem. 298, 102061. 10.1016/j.jbc.2022.102061 35609712 PMC9218512

[B9] de Juan-Sanz J. Holt G. T. Schreiter E. R. de Juan F. Kim D. S. Ryan T. A. (2017). Axonal endoplasmic reticulum Ca^2+^ content controls release probability in CNS nerve terminals. Neuron 93 (4), 867–881. 10.1016/j.neuron.2017.01.010 28162809 PMC5325711

[B11] Dupont G. Combettes L. Bird G. S. Putney J. W. (2011). Calcium oscillations. Cold Spring Harb. Perspect. Biol. 3 (3), a004226. 10.1101/cshperspect.a004226 21421924 PMC3039928

[B12] Flourakis M. Van Coppenolle F. Lehen'kyi V. Beck B. Skryma R. Prevarskaya N. (2006). Passive calcium leak via translocon is a first step for iPLA2-pathway regulated store operated channels activation. FASEB J. official Publ. Fed. Am. Soc. Exp. Biol. 20 (8), 1215–1217. 10.1096/fj.05-5254fje 16611832

[B13] Garbincius J. F. Elrod J. W. (2022). Mitochondrial calcium exchange in physiology and disease. Physiol. Rev. 102 (2), 893–992. 10.1152/physrev.00041.2020 34698550 PMC8816638

[B14] Hartmann J. Karl R. M. Alexander R. P. Adelsberger H. Brill M. S. Ruhlmann C. (2014). STIM1 controls neuronal Ca²⁺ signaling, mGluR1-dependent synaptic transmission, and cerebellar motor behavior. Neuron 82 (3), 635–644. 10.1016/j.neuron.2014.03.027 24811382

[B15] Hasel P. Dando O. Jiwaji Z. Baxter P. Todd A. C. Heron S. (2017). Neurons and neuronal activity control gene expression in astrocytes to regulate their development and metabolism. Nat. Commun. 8, 15132. 10.1038/ncomms15132 28462931 PMC5418577

[B16] Hofer A. M. Machen T. E. (1993). Technique for *in situ* measurement of calcium in intracellular inositol 1, 4, 5-trisphosphate-sensitive stores using the fluorescent indicator mag-fura-2. Proc. Natl. Acad. Sci. U. S. A. 90 (7), 2598–2602. 10.1073/pnas.90.7.2598 8464866 PMC46142

[B17] Kipanyula M. J. Contreras L. Zampese E. Lazzari C. Wong A. K. Pizzo P. (2012). Ca^2+^ dysregulation in neurons from transgenic mice expressing mutant presenilin 2. Aging Cell 11 (5), 885–893. 10.1111/j.1474-9726.2012.00858.x 22805202

[B18] Ishii Kiyoaki Hirose Kenzo Iino Masamitsu (2006). Ca^2+^ shuttling between endoplasmic reticulum and mitochondria underlying Ca^2+^ oscillations. EMBO Rep. 7 (4), 390–396. 10.1038/sj.embor.7400620 16415789 PMC1456907

[B19] Lalonde J. Saia G. Gill G. (2014). Store-operated calcium entry promotes the degradation of the transcription factor Sp4 in resting neurons. Sci. Signal. 7 (328), ra51. 10.1126/scisignal.2005242 24894994 PMC4445882

[B20] Lang S. Pfeffer S. Lee P. H. Cavalie A. Helms V. Forster F. (2017). An update on Sec61 channel functions, mechanisms, and related diseases. Front. Physiol. 8, 887. 10.3389/fphys.2017.00887 29163222 PMC5672155

[B21] Laude A. J. Tovey S. C. Dedos S. G. Potter B. V. Lummis S. C. Taylor C. W. (2005). Rapid functional assays of recombinant IP3 receptors. Cell calcium 38 (1), 45–51. 10.1016/j.ceca.2005.04.001 15963563

[B23] Lemos F. O. Bultynck G. Parys J. B. (2021). A comprehensive overview of the complex world of the endo- and sarcoplasmic reticulum Ca(2+)-leak channels. Biochim. Biophys. Acta. Mol. Cell Res. 1868 (7), 119020. 10.1016/j.bbamcr.2021.119020 33798602

[B24] Lim D. Semyanov A. Genazzani A. Verkhratsky A. (2021). Calcium signaling in neuroglia. Int. Rev. Cell Mol. Biol. 362, 1–53. 10.1016/bs.ircmb.2021.01.003 34253292

[B25] Mekahli D. Bultynck G. Parys J. B. De Smedt H. Missiaen L. (2011). Endoplasmic-reticulum calcium depletion and disease. Cold Spring Harb. Perspect. Biol. 3 (6), a004317. 10.1101/cshperspect.a004317 21441595 PMC3098671

[B27] Petersen O. H. Courjaret R. Machaca K. (2017). Ca(2+) tunnelling through the ER lumen as a mechanism for delivering Ca(2+) entering via store-operated Ca(2+) channels to specific target sites. J. Physiol. 595 (10), 2999–3014. 10.1113/JP272772 28181236 PMC5430212

[B29] Prada J. Sasi M. Martin C. Jablonka S. Dandekar T. Blum R. (2018). An open source tool for automatic spatiotemporal assessment of calcium transients and local 'signal-close-to-noise' activity in calcium imaging data. PLoS Comput. Biol. 14 (3), e1006054. 10.1371/journal.pcbi.1006054 29601577 PMC5895056

[B30] Putney J. W. Steinckwich-Besancon N. Numaga-Tomita T. Davis F. M. Desai P. N. D'Agostin D. M. (2017). The functions of store-operated calcium channels. Biochim. Biophys. Acta. Mol. Cell Res. 1864 (6), 900–906. 10.1016/j.bbamcr.2016.11.028 27913208 PMC5420336

[B31] Rehberg M. Lepier A. Solchenberger B. Osten P. Blum R. (2008). A new non-disruptive strategy to target calcium indicator dyes to the endoplasmic reticulum. Cell calcium 44 (4), 386–399. 10.1016/j.ceca.2008.02.002 19230142

[B32] Rodriguez-Garcia A. Rojo-Ruiz J. Navas-Navarro P. Aulestia F. J. Gallego-Sandin S. Garcia-Sancho J. (2014). GAP, an aequorin-based fluorescent indicator for imaging Ca^2+^ in organelles. Proc. Natl. Acad. Sci. U. S. A. 111 (7), 2584–2589. 10.1073/pnas.1316539111 24501126 PMC3932923

[B33] Rodríguez-Prados M. Rojo-Ruiz J. García-Sancho J. Alonso M. T. (2020). Direct monitoring of ER Ca^2+^ dynamics reveals that Ca^2+^ entry induces ER-Ca^2+^ release in astrocytes. Pflugers Arch. 472 (4), 439–448. 10.1007/s00424-020-02364-7 32246199

[B34] Rodriguez-Prados M. Rojo-Ruiz J. Garcia-Sancho J. Alonso M. T. (2020). Direct monitoring of ER Ca(2+) dynamics reveals that Ca(2+) entry induces ER-Ca(2+) release in astrocytes. Pflugers Arch. 472 (4), 439–448. 10.1007/s00424-020-02364-7 32246199

[B35] Rossi A. M. Taylor C. W. (2020). Reliable measurement of free Ca(2+) concentrations in the ER lumen using Mag-Fluo-4. Cell calcium 87, 102188. 10.1016/j.ceca.2020.102188 32179239 PMC7181174

[B10] Stefani Diego De Raffaello Anna Teardo Enrico Szabò Ildikò Rizzuto Rosario (2011). A forty-kilodalton protein of the inner membrane is the mitochondrial calcium uniporter. Nature 476 (7360), 336–340. 10.1038/nature10230 21685888 PMC4141877

[B36] Samtleben S. Jaepel J. Fecher C. Andreska T. Rehberg M. Blum R. (2013). Direct imaging of ER calcium with targeted-esterase induced dye loading (TED). J. Vis. Exp. 75 (75), e50317. 10.3791/50317 PMC3679584 23685703

[B37] Samtleben S. Wachter B. Blum R. (2015). Store-operated calcium entry compensates fast ER calcium loss in resting hippocampal neurons. Cell calcium 58 (2), 147–159. 10.1016/j.ceca.2015.04.002 25957620

[B38] Satoh K. Matsu-ura T. Enomoto M. Nakamura H. Michikawa T. Mikoshiba K. (2011). Highly cooperative dependence of sarco/endoplasmic reticulum calcium ATPase (SERCA) 2a pump activity on cytosolic calcium in living cells. J. Biol. Chem. 286 (23), 20591–20599. 10.1074/jbc.M110.204685 21515674 PMC3121519

[B39] Schäuble N. Lang S. Jung M. Cappel S. Schorr S. Ulucan O. (2012). BiP-mediated closing of the Sec61 channel limits Ca^2+^ leakage from the ER. EMBO J. 31 (15), 3282–3296. 10.1038/emboj.2012.189 22796945 PMC3411083

[B40] Schulte A. Bieniussa L. Gupta R. Samtleben S. Bischler T. Doering K. (2022). Homeostatic calcium fluxes, ER calcium release, SOCE, and calcium oscillations in cultured astrocytes are interlinked by a small calcium toolkit. Cell calcium 101, 102515. 10.1016/j.ceca.2021.102515 34896701

[B41] Solovyova N. Verkhratsky A. (2002). Monitoring of free calcium in the neuronal endoplasmic reticulum: An overview of modern approaches. J. Neurosci. Methods 122 (1), 1–12. 10.1016/s0165-0270(02)00300-x 12535760

[B42] Solovyova N. Veselovsky N. Toescu E. C. Verkhratsky A. (2002). Ca(2+) dynamics in the lumen of the endoplasmic reticulum in sensory neurons: Direct visualization of Ca(2+)-induced Ca(2+) release triggered by physiological Ca(2+) entry. EMBO J. 21 (4), 622–630. 10.1093/emboj/21.4.622 11847110 PMC125857

[B43] Streb H. Irvine R. F. Berridge M. J. Schulz I. (1983). Release of Ca^2+^ from a nonmitochondrial intracellular store in pancreatic acinar cells by inositol-1, 4, 5-trisphosphate. Nature 306 (5938), 67–69. 10.1038/306067a0 6605482

[B44] Suzuki J. Kanemaru K. Ishii K. Ohkura M. Okubo Y. Iino M. (2014). Imaging intraorganellar Ca^2+^ at subcellular resolution using CEPIA. Nat. Commun. 5, 4153. 10.1038/ncomms5153 24923787 PMC4082642

[B45] Tang S. Wong H-C. Wang Z-M. Huang Y. Zou J. Zhuo Y. (2011). Design and application of a class of sensors to monitor Ca^2+^ dynamics in high Ca^2+^ concentration cellular compartments. Proc. Natl. Acad. Sci. U. S. A. 108 (39), 16265–16270. 10.1073/pnas.1103015108 21914846 PMC3182728

[B46] Thastrup O. Dawson A. P. Scharff O. Foder B. Cullen P. J. Drobak B. K. (1989). Thapsigargin, a novel molecular probe for studying intracellular calcium release and storage. Agents Actions 27 (1-2), 17–23. 10.1007/BF02222186 2787587

[B47] Verkhratsky A. Nedergaard M. (2018). Physiology of astroglia. Physiol. Rev. 98 (1), 239–389. 10.1152/physrev.00042.2016 29351512 PMC6050349

[B48] Verkhratsky A. (2005). Physiology and pathophysiology of the calcium store in the endoplasmic reticulum of neurons. Physiol. Rev. 85 (1), 201–279. 10.1152/physrev.00004.2004 15618481

[B26] Young Michael Schug Zachary T. Booth David Yule David Mikoshiba Katsuhiko Hajnoczky Gyorgy (2022). Metabolic adaptation to the chronic loss of Ca(2+) signaling induced by KO of IP3 receptors or the mitochondrial Ca(2+) uniporter. J. Biol. Chem. 298 (1), 101436. 10.1016/j.jbc.2021.101436 34801549 PMC8672050

